# The genome sequence of *a *picture-winged fly *, Melieria omissa* (Meigen, 1826) (Diptera: Ulidiidae)

**DOI:** 10.12688/wellcomeopenres.24956.1

**Published:** 2025-10-03

**Authors:** Erica McAlister, Ryan Mitchell, Olga Sivell, Natural History Museum Genome Acquisition Lab, Darwin Tree of Life Barcoding Collective, Wellcome Sanger Institute Tree of Life Management, Samples and Laboratory team, Wellcome Sanger Institute Scientific Operations: Sequencing Operations, Wellcome Sanger Institute Tree of Life Core Informatics team, Tree of Life Core Informatics collective, Darwin Tree of Life Consortium

**Affiliations:** 1 Natural History Museum, London, England, UK; 2 Independent researcher, Sligo, County Sligo, Ireland

**Keywords:** Melieria omissa; picture-winged fly; genome sequence; chromosomal; Diptera

## Abstract

We present a genome assembly from an individual female *Melieria omissa* (Arthropoda; Insecta; Diptera; Ulidiidae). The genome sequence has a total length of 490.54 megabases. Most of the assembly (97.17%) is scaffolded into 4 chromosomal pseudomolecules. The mitochondrial genome has also been assembled, with a length of 15.76 kilobases. Gene annotation of this assembly on Ensembl identified 21 533 protein-coding genes. This assembly was generated as part of the Darwin Tree of Life project, which produces reference genomes for eukaryotic species found in Britain and Ireland.

## Species taxonomy

Eukaryota; Opisthokonta; Metazoa; Eumetazoa; Bilateria; Protostomia; Ecdysozoa; Panarthropoda; Arthropoda; Mandibulata; Pancrustacea; Hexapoda; Insecta; Dicondylia; Pterygota; Neoptera; Endopterygota; Diptera; Brachycera; Muscomorpha; Eremoneura; Cyclorrhapha; Schizophora; Acalyptratae; Tephritoidea; Ulidiidae; Otitinae; *Melieria*; *Melieria omissa* (Meigen, 1826) (NCBI:txid1262360)

## Background

*Melieria omissa* (Meigen, 1826) is a picture-winged fly from the family Ulidiidae (and previously in Otitidae which is now falls within Ulidiidae). There are four species of *Melieria* Robineau-Desvoidy, 1830 that are recorded from Britain, two of these are smaller species (body up to 5 mm long): *M. cana* (Loew, 1858) and *M. picta* (Meigen, 1826), and two medium-sized (body 6 mm or longer): *M. omissa* and *M. crassipennis* (Fabricius, 1794) ( Chandler, 2025; Clements, 1990; Clements, 2020).

*Melieria omissa* is a characteristic fly with greyish body, unicoloured abdominal tergites (dark posterior margins are present in the similar-looking *M. crassipennis*) and the first flagellomere (postpedicel) of the antenna is pointed; wings appear orange from certain angles and are adorned with large black spots, the basal spot of *M. omissa* does not extend to the costal cell (unlike in *M. crassipennis*); the frons is broad and orange, the femora are pale or narrowly darkened, while broadly darkened in the other *Melieria* species ( Clements, 1990; Clements, 2020; Greve, 1988; Smit, 2010).

This is a Palaearctic species with a predominantly coastal distribution ( GBIF Secretariat, 2023; NBN Atlas Partnership, 2025; Smit, 2010). Smit (2010) noted that inland records in the Netherlands are sporadic and of single individuals which suggested they are vagrant or carried by wind. However, a consistency of inland records, such as in the Cambridgeshire Fens, shows that populations of *M. omissa* are not restricted to coastal locations in Britain ( NBN Atlas Partnership, 2025). The species is scarce, but widespread in England and Wales, with no confirmed occurrences from Scotland and absent from Ireland ( Chandler, 2025; Clements, 2020; NBN Atlas Partnership, 2025; Speight & Chandler, 1983). The immature stages remain unknown ( Smith, 1989). In Britain, the adults have been reported from vegetation bordering ponds and streams, e.g. Yellow Flag and Water Hemlock ( Colyer & Hammond, 1951). Smit (2010) notes the larvae likely develop in reeds stems, in *Phragmites australis* according to Kabos & Van Aartsen (1984). In Britain the adults are on the wing from May to August, peaking June to July ( NBN Atlas Partnership, 2025).

**Figure 1.**
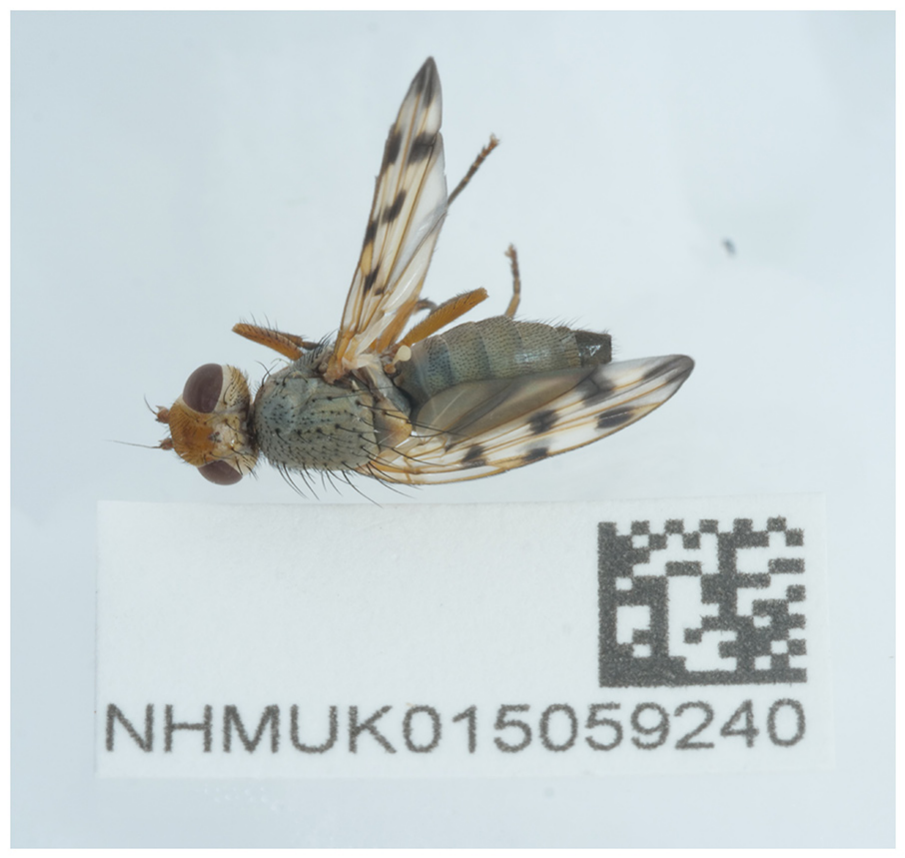
Photograph of the *Melieria omissa* (idMelOmis1) specimen used for genome sequencing.

The high-quality genome of *Melieria omissa* was sequenced from a single specimen (NHMUK015059240; SAMEA112963096) from Berney Marshes, England ( Figure 1). The genome of *M. omissa* presented here was sequenced as part of the Darwin Tree of Life Project, a collaborative effort to sequence all named eukaryotic species in the Atlantic Archipelago of Britain and Ireland. The present assembly is one of three genomes for the genus *Melieria* as of August 2025 (data obtained via NCBI datasets, O’Leary *et al.*, 2024), and is the RefSeq reference assembly for this species. Genomes *Melieria* have been published by the Darwin Tree of Life Project, namely *M. picta* and *M. crassipennis* ( Mitchell *et al.*, 2024). They will aid research into phylogeny of Ulidiidae and Tephritoidea, as well as taxonomy and biology of the species.

## Methods

### Sample acquisition and DNA barcoding

The specimen used for genome sequencing was an adult female *Melieria omissa* (specimen ID NHMUK015059240, ToLID idMelOmis1; Figure 1), collected from the RSPB Reserve, Berney Marshes, England, UK (latitude 52.59, longitude 1.64) on 2022-07-05. The specimen was collected by Erica McAlister and formally identified by Ryan Mitchell. For the Darwin Tree of Life sampling and metadata approach, refer to Lawniczak *et al*. (2022).

The initial identification was verified by an additional DNA barcoding process according to the framework developed by Twyford *et al.* (2024). A small sample was dissected from the specimen and stored in ethanol, while the remaining parts were shipped on dry ice to the Wellcome Sanger Institute (WSI) (see the protocol). The tissue was lysed, the COI marker region was amplified by PCR, and amplicons were sequenced and compared to the BOLD database, confirming the species identification ( Crowley *et al.*, 2023). Following whole genome sequence generation, the relevant DNA barcode region was also used alongside the initial barcoding data for sample tracking at the WSI ( Twyford *et al.*, 2024). The standard operating procedures for Darwin Tree of Life barcoding are available on protocols.io.

### Nucleic acid extraction

Protocols for high molecular weight (HMW) DNA extraction developed at the Wellcome Sanger Institute (WSI) Tree of Life Core Laboratory are available on protocols.io ( Howard *et al.*, 2025). The idMelOmis1 sample was weighed and triaged to determine the appropriate extraction protocol. Tissue from the whole organism was homogenised by powermashing using a PowerMasher II tissue disruptor.

HMW DNA was extracted in the WSI Scientific Operations core using the Automated MagAttract v2 protocol. DNA was sheared into an average fragment size of 12–20 kb following the Megaruptor®3 for LI PacBio protocol. Sheared DNA was purified by manual SPRI (solid-phase reversible immobilisation). The concentration of the sheared and purified DNA was assessed using a Nanodrop spectrophotometer and Qubit Fluorometer using the Qubit dsDNA High Sensitivity Assay kit. Fragment size distribution was evaluated by running the sample on the FemtoPulse system. For this sample, the final post-shearing DNA had a Qubit concentration of 20.14 ng/μL and a yield of 926.44 ng, with a fragment size of 11.8 kb. The 260/280 spectrophotometric ratio was 1.96, and the 260/230 ratio was 2.92.

### PacBio HiFi library preparation and sequencing

Library preparation and sequencing were performed at the WSI Scientific Operations core. Libraries were prepared using the SMRTbell Prep Kit 3.0 (Pacific Biosciences, California, USA), following the manufacturer’s instructions. The kit includes reagents for end repair/A-tailing, adapter ligation, post-ligation SMRTbell bead clean-up, and nuclease treatment. Size selection and clean-up were performed using diluted AMPure PB beads (Pacific Biosciences). DNA concentration was quantified using a Qubit Fluorometer v4.0 (ThermoFisher Scientific) and the Qubit 1X dsDNA HS assay kit. Final library fragment size was assessed with the Agilent Femto Pulse Automated Pulsed Field CE Instrument (Agilent Technologies) using the gDNA 55 kb BAC analysis kit.

The sample was sequenced on a Revio instrument (Pacific Biosciences). The prepared library was normalised to 2 nM, and 15 μL was used for making complexes. Primers were annealed and polymerases bound to generate circularised complexes, following the manufacturer’s instructions. Complexes were purified using 1.2X SMRTbell beads, then diluted to the Revio loading concentration (200–300 pM) and spiked with a Revio sequencing internal control. The sample was sequenced on a Revio 25M SMRT cell. The SMRT Link software (Pacific Biosciences), a web-based workflow manager, was used to configure and monitor the run and to carry out primary and secondary data analysis.

### Hi-C

***Sample preparation and crosslinking***

The Hi-C sample was prepared from 20–50 mg of frozen tissue of the idMelOmis1 sample using the Arima-HiC v2 kit (Arima Genomics). Following the manufacturer’s instructions, tissue was fixed and DNA crosslinked using TC buffer to a final formaldehyde concentration of 2%. The tissue was homogenised using the Diagnocine Power Masher-II. Crosslinked DNA was digested with a restriction enzyme master mix, biotinylated, and ligated. Clean-up was performed with SPRISelect beads before library preparation. DNA concentration was measured with the Qubit Fluorometer (Thermo Fisher Scientific) and Qubit HS Assay Kit. The biotinylation percentage was estimated using the Arima-HiC v2 QC beads.

***Hi-C library preparation and sequencing***

Biotinylated DNA constructs were fragmented using a Covaris E220 sonicator and size selected to 400–600 bp using SPRISelect beads. DNA was enriched with Arima-HiC v2 kit Enrichment beads. End repair, A-tailing, and adapter ligation were carried out with the NEBNext Ultra II DNA Library Prep Kit (New England Biolabs), following a modified protocol where library preparation occurs while DNA remains bound to the Enrichment beads. Library amplification was performed using KAPA HiFi HotStart mix and a custom Unique Dual Index (UDI) barcode set (Integrated DNA Technologies). Depending on sample concentration and biotinylation percentage determined at the crosslinking stage, libraries were amplified with 10 to 16 PCR cycles. Post-PCR clean-up was performed with SPRISelect beads. Libraries were quantified using the AccuClear Ultra High Sensitivity dsDNA Standards Assay Kit (Biotium) and a FLUOstar Omega plate reader (BMG Labtech).

Prior to sequencing, libraries were normalised to 10 ng/μL. Normalised libraries were quantified again and equimolar and/or weighted 2.8 nM pools. Pool concentrations were checked using the Agilent 4200 TapeStation (Agilent) with High Sensitivity D500 reagents before sequencing. Sequencing was performed using paired-end 150 bp reads on the Illumina NovaSeq 6000.

### Genome assembly

Prior to assembly of the PacBio HiFi reads, a database of *k*-mer counts ( *k* = 31) was generated from the filtered reads using FastK. GenomeScope2 ( Ranallo-Benavidez *et al.*, 2020) was used to analyse the *k*-mer frequency distributions, providing estimates of genome size, heterozygosity, and repeat content.

The HiFi reads were assembled using Hifiasm ( Cheng *et al.*, 2021) with the --primary option. The Hi-C reads ( Rao *et al.*, 2014) were mapped to the primary contigs using bwa-mem2 ( Vasimuddin *et al.*, 2019), and the contigs were scaffolded in YaHS ( Zhou *et al.*, 2023) with the --break option for handling potential misassemblies. The scaffolded assemblies were evaluated using Gfastats ( Formenti *et al.*, 2022), BUSCO ( Manni *et al.*, 2021) and MERQURY.FK ( Rhie *et al.*, 2020).

The mitochondrial genome was assembled using MitoHiFi ( Uliano-Silva *et al.*, 2023), which runs MitoFinder ( Allio *et al.*, 2020) and uses these annotations to select the final mitochondrial contig and to ensure the general quality of the sequence.

### Assembly curation

The assembly was decontaminated using the Assembly Screen for Cobionts and Contaminants ( ASCC) pipeline. TreeVal was used to generate the flat files and maps for use in curation. Manual curation was conducted primarily in PretextView and HiGlass ( Kerpedjiev *et al.*, 2018). Scaffolds were visually inspected and corrected as described by Howe *et al*. (2021). Manual corrections included 16 breaks, 40 joins, and removal of 18 haplotypic duplications. The curation process is documented at https://gitlab.com/wtsi-grit/rapid-curation. PretextSnapshot was used to generate a Hi-C contact map of the final assembly.

### Assembly quality assessment

The Merqury.FK tool ( Rhie *et al.*, 2020) was run in a Singularity container ( Kurtzer *et al.*, 2017) to evaluate *k*-mer completeness and assembly quality for the primary and alternate haplotypes using the *k*-mer databases ( *k* = 31) computed prior to genome assembly. The analysis outputs included assembly QV scores and completeness statistics.

The genome was analysed using the BlobToolKit pipeline, a Nextflow implementation of the earlier Snakemake version ( Challis *et al.*, 2020). The pipeline aligns PacBio reads using minimap2 ( Li, 2018) and SAMtools ( Danecek *et al.*, 2021) to generate coverage tracks. It runs BUSCO ( Manni *et al.*, 2021) using lineages identified from the NCBI Taxonomy ( Schoch *et al.*, 2020). For the three domain-level lineages, BUSCO genes are aligned to the UniProt Reference Proteomes database ( Bateman *et al.*, 2023) using DIAMOND blastp ( Buchfink *et al.*, 2021). The genome is divided into chunks based on the density of BUSCO genes from the closest taxonomic lineage, and each chunk is aligned to the UniProt Reference Proteomes database with DIAMOND blastx. Sequences without hits are chunked using seqtk and aligned to the NT database with blastn ( Altschul *et al.*, 1990). The BlobToolKit suite consolidates all outputs into a blobdir for visualisation. The BlobToolKit pipeline was developed using nf-core tooling ( Ewels *et al.*, 2020) and MultiQC ( Ewels *et al.*, 2016), with containerisation through Docker ( Merkel, 2014) and Singularity ( Kurtzer *et al.*, 2017).

## Genome sequence report

### Sequence data

**Figure 2.**
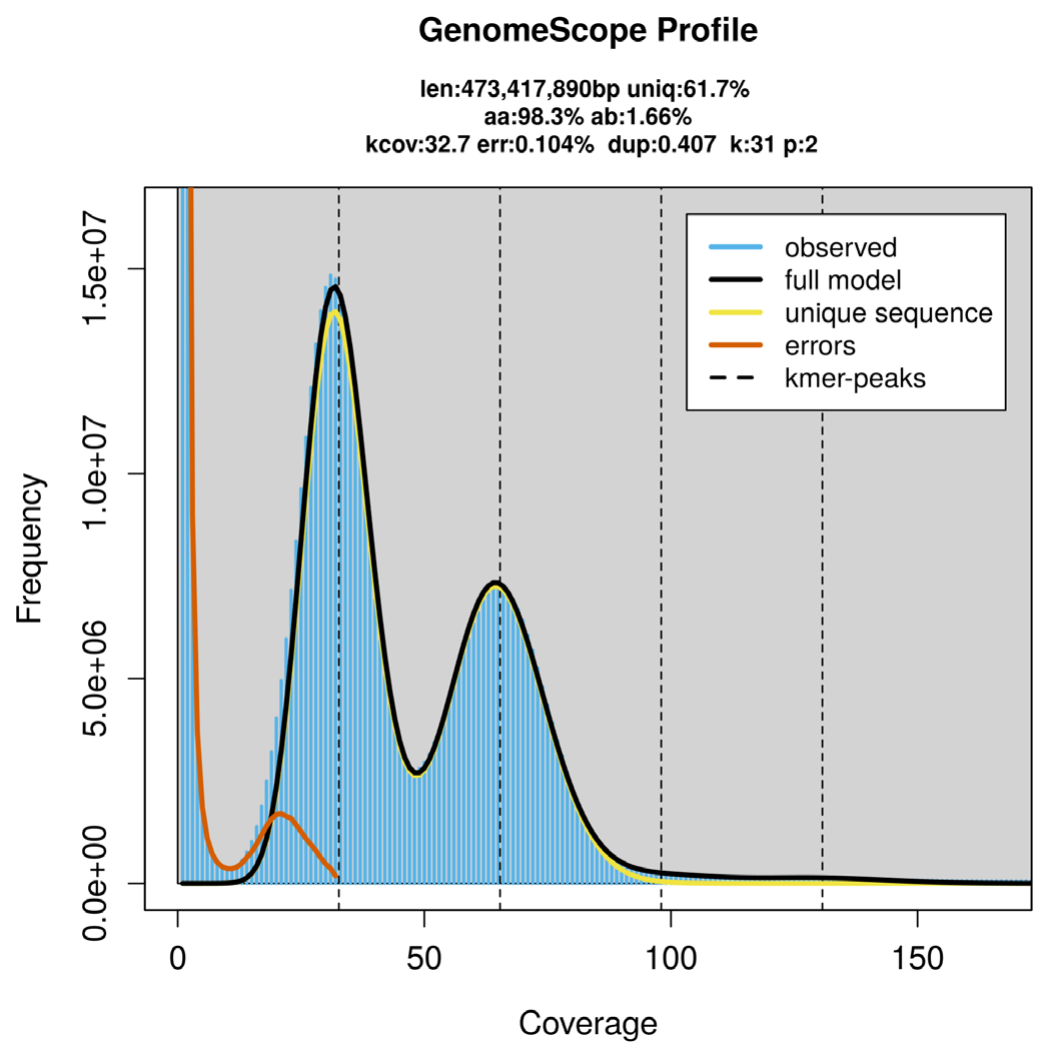
Frequency distribution of *k*-mers generated using GenomeScope2. The plot shows observed and modelled *k*-mer spectra, providing estimates of genome size, heterozygosity, and repeat content based on unassembled sequencing reads.

PacBio sequencing of the *Melieria omissa* specimen generated 32.05 Gb (gigabases) from 3.28 million reads, which were used to assemble the genome. GenomeScope2.0 analysis estimated the haploid genome size at 473.42 Mb, with a heterozygosity of 1.66% and repeat content of 38.40% ( Figure 2). These estimates guided expectations for the assembly. Based on the estimated genome size, the sequencing data provided approximately 65× coverage. Hi-C sequencing produced 110.12 Gb from 729.27 million reads, which were used to scaffold the assembly. Table 1 summarises the specimen and sequencing details.

**Table 1.** Specimen and sequencing data for BioProject PRJEB67433.

Platform	PacBio HiFi	Hi-C
**ToLID**	idMelOmis1	idMelOmis1
**Specimen ID**	NHMUK015059240	NHMUK015059240
**BioSample (source individual)**	SAMEA112963096	SAMEA112963096
**BioSample (tissue)**	SAMEA112963192	SAMEA112963192
**Tissue**	whole organism	whole organism
**Instrument**	Revio	Illumina NovaSeq 6000
**Run accessions**	ERR12120054	ERR12121882
**Read count total**	3.28 million	729.27 million
**Base count total**	32.05 Gb	110.12 Gb

### Assembly statistics

The primary haplotype was assembled, and contigs corresponding to an alternate haplotype were also deposited in INSDC databases. The final assembly has a total length of 490.54 Mb in 173 scaffolds, with 177 gaps, and a scaffold N50 of 127.74 Mb ( Table 2).

**Table 2.** Genome assembly statistics.

**Assembly name**	idMelOmis1.1
**Assembly accession**	GCA_963971225.1
**Alternate haplotype accession**	GCA_963971195.1
**Assembly level**	chromosome
**Span (Mb)**	490.54
**Number of chromosomes**	4
**Number of contigs**	350
**Contig N50**	4.09 Mb
**Number of scaffolds**	173
**Scaffold N50**	127.74 Mb
**Sex chromosomes**	not identified
**Organelles**	Mitochondrion: 15.76 kb

**Figure 3.**
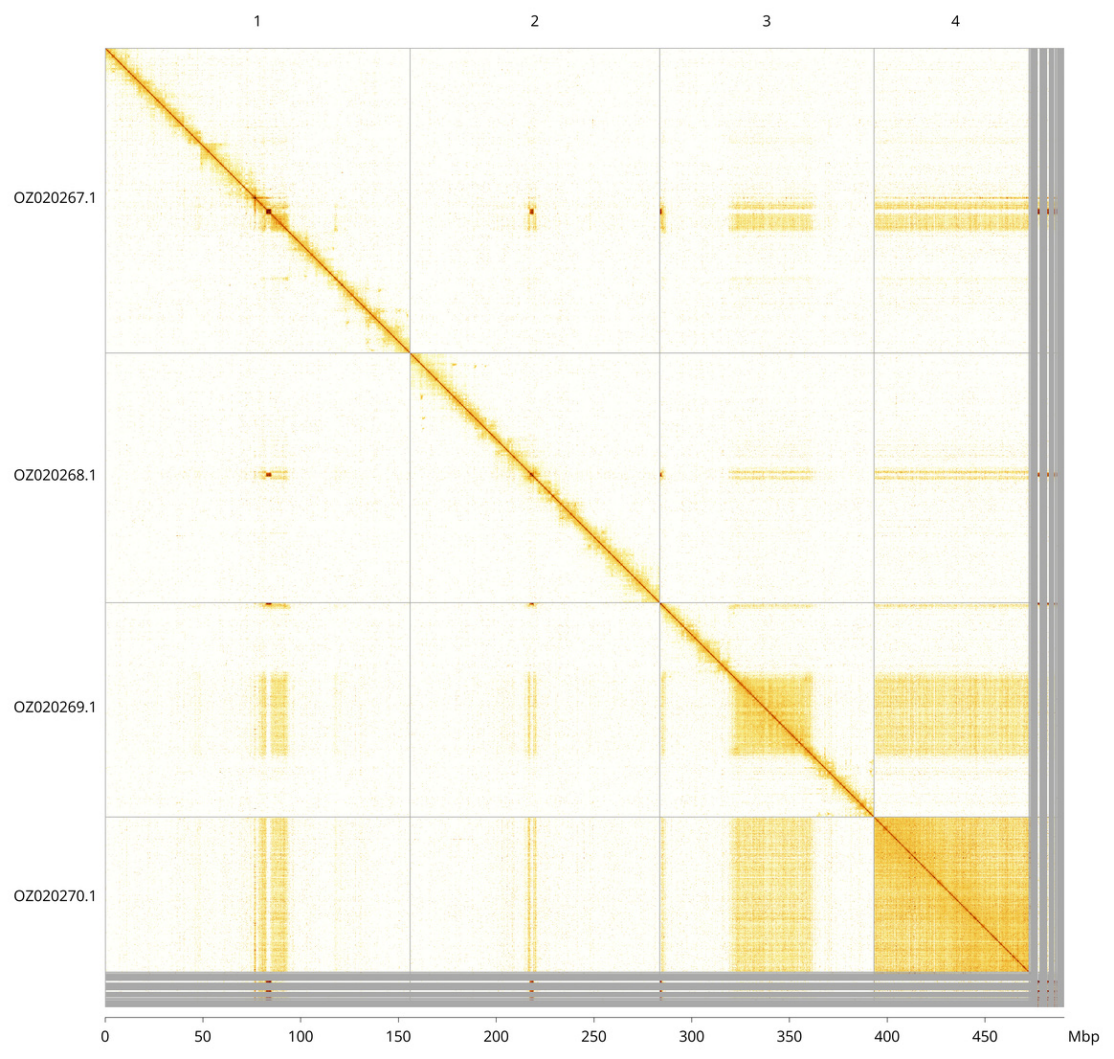
Hi-C contact map of the *Melieria omissa* genome assembly. Assembled chromosomes are shown in order of size and labelled along the axes, with a megabase scale shown below. The plot was generated using PretextSnapshot.

Most of the assembly sequence (97.17%) was assigned to 4 chromosomal-level scaffolds. These chromosome-level scaffolds, confirmed by Hi-C data, are named according to size ( Figure 3; Table 3). We did not identify the sex chromosome(s) as sequence data from the heterogametic sex was not available, and homology is unreliable for sex chromosome identification in Diptera due to frequent sex chromosome turnover ( Vicoso & Bachtrog, 2015).

**Table 3.** Chromosomal pseudomolecules in the primary genome assembly of *Melieria omissa* idMelOmis1.

INSDC accession	Molecule	Length (Mb)	GC%
OZ020267.1	1	155.96	38.50
OZ020268.1	2	127.74	38
OZ020269.1	3	109.51	39
OZ020270.1	4	83.47	40.50

The mitochondrial genome was also assembled. This sequence is included as a contig in the multifasta file of the genome submission and as a standalone record.

**Figure 4.**
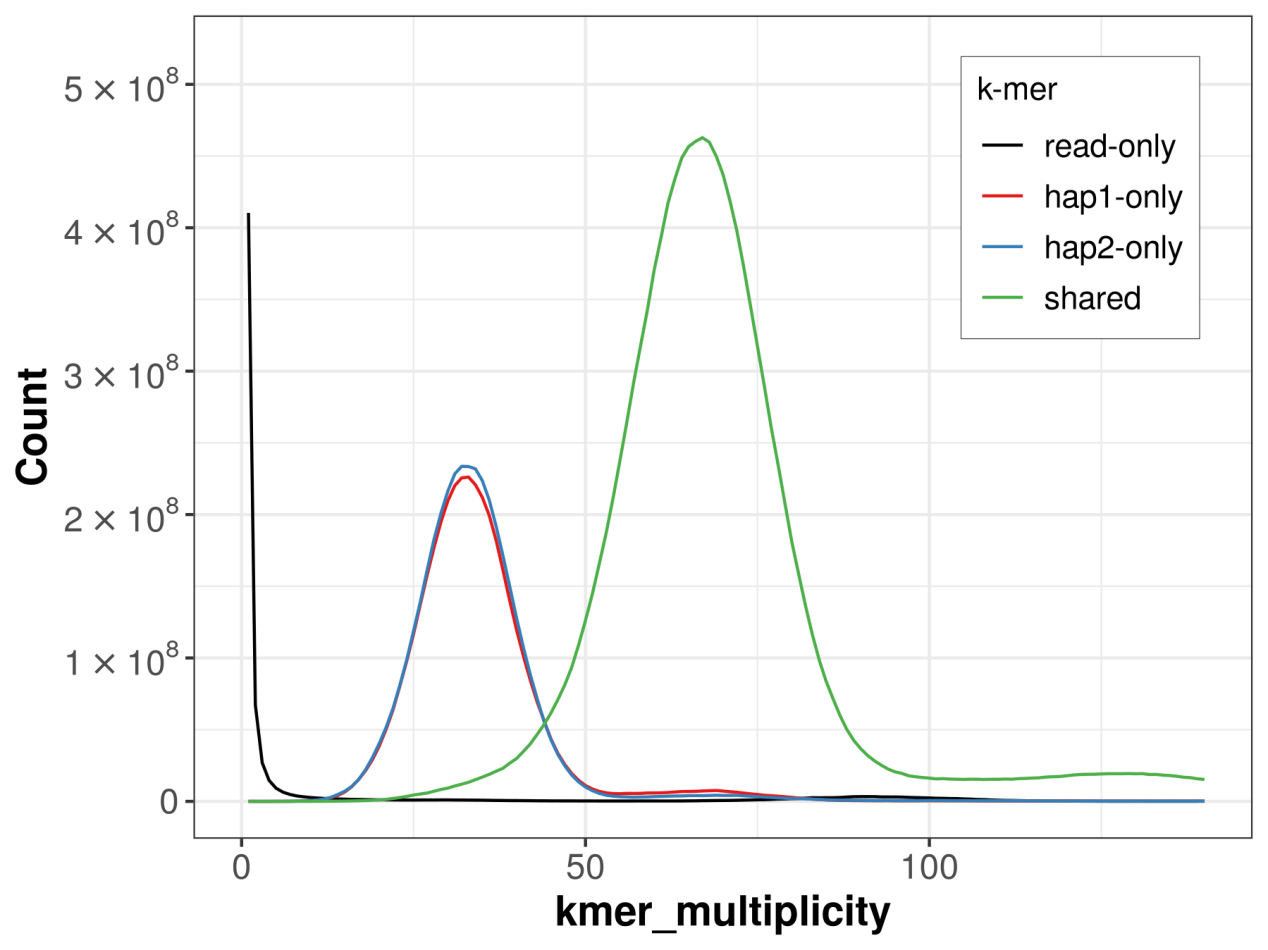
Evaluation of *k*-mer completeness using MerquryFK. This plot illustrates the recovery of *k*-mers from the original read data in the final assemblies. The horizontal axis represents *k*-mer multiplicity, and the vertical axis shows the number of *k*-mers. The black curve represents *k*-mers that appear in the reads but are not assembled. The green curve corresponds to *k*-mers shared by both haplotypes, and the red and blue curves show *k*-mers found only in one of the haplotypes.

The combined primary and alternate assemblies achieve an estimated QV of 58.5. The *k*-mer completeness is 71.47% for the primary assembly, 72.19% for the alternate haplotype, and 99.11% for the combined assemblies ( Figure 4).

**Figure 5.**
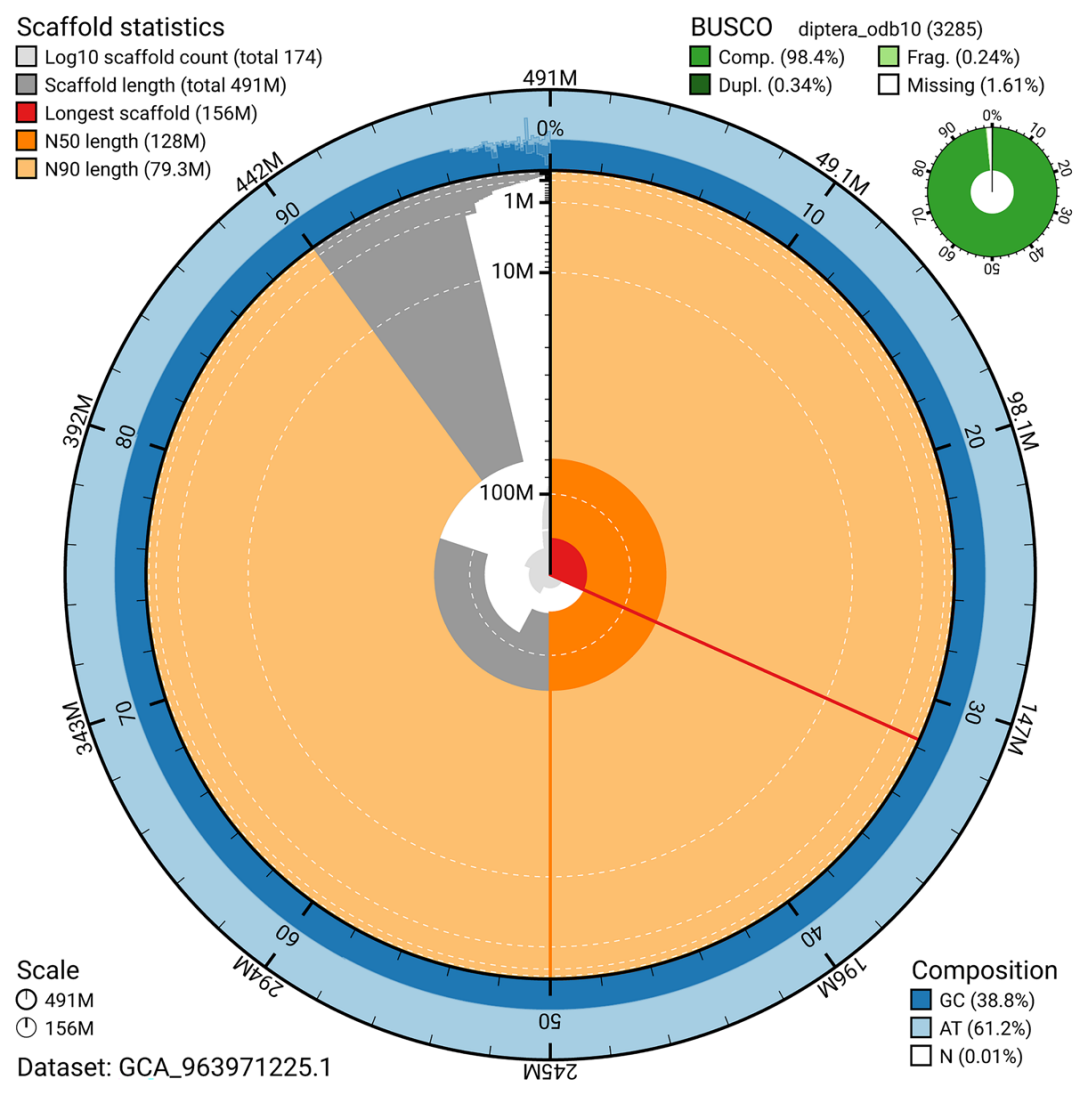
Assembly metrics for idMelOmis1.1. The BlobToolKit snail plot provides an overview of assembly metrics and BUSCO gene completeness. The circumference represents the length of the whole genome sequence, and the main plot is divided into 1 000 bins around the circumference. The outermost blue tracks display the distribution of GC, AT, and N percentages across the bins. Scaffolds are arranged clockwise from longest to shortest and are depicted in dark grey. The longest scaffold is indicated by the red arc, and the deeper orange and pale orange arcs represent the N50 and N90 lengths. A light grey spiral at the centre shows the cumulative scaffold count on a logarithmic scale. A summary of complete, fragmented, duplicated, and missing BUSCO genes in the diptera_odb10 set is presented at the top right. An interactive version of this figure can be accessed on the BlobToolKit viewer.

**Figure 6.**
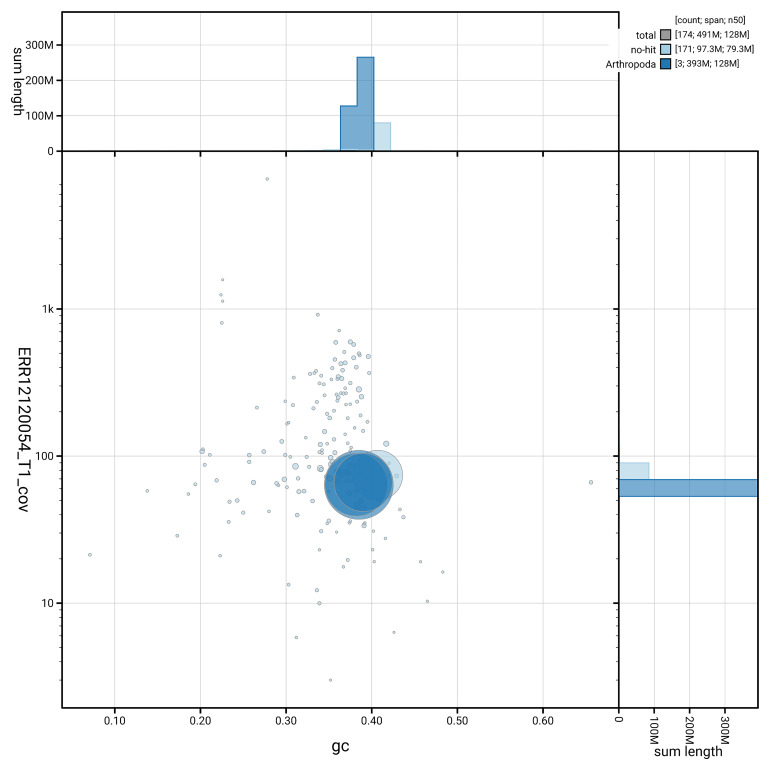
BlobToolKit GC-coverage plot for idMelOmis1.1. Blob plot showing sequence coverage (vertical axis) and GC content (horizontal axis). The circles represent scaffolds, with the size proportional to scaffold length and the colour representing phylum membership. The histograms along the axes display the total length of sequences distributed across different levels of coverage and GC content. An interactive version of this figure is available on the BlobToolKit viewer.

BUSCO v.5.5.0 analysis using the diptera_odb10 reference set ( *n* = 3 285) identified 98.4% of the expected gene set (single = 98.1%, duplicated = 0.3%). The snail plot in Figure 5 summarises the scaffold length distribution and other assembly statistics for the primary assembly. The blob plot in Figure 6 shows the distribution of scaffolds by GC proportion and coverage.

Table 4 lists the assembly metric benchmarks adapted from Rhie *et al.* (2021) the Earth BioGenome Project Report on Assembly Standards September 2024. The EBP metric, calculated for the primary assembly, is **6.C.Q58**, meeting the recommended reference standard.

**Table 4.** Earth Biogenome Project summary metrics for the *Melieria omissa* assembly.

Measure	Value	Benchmark
EBP summary (primary)	6.C.Q58	6.C.Q40
Contig N50 length	4.09 Mb	≥ 1 Mb
Scaffold N50 length	127.74 Mb	= chromosome N50
Consensus quality (QV)	Primary: 58.3; alternate: 58.8; combined: 58.5	≥ 40
*k*-mer completeness	Primary: 71.47%; alternate: 72.19%; combined: 99.11%	≥ 95%
BUSCO	C:98.4% [S:98.1%; D:0.3%]; F:0.2%; M:1.4%; n:3 285	S > 90%; D < 5%
Percentage of assembly assigned to chromosomes	97.17%	≥ 90%

## Genome annotation report

The *Melieria omissa* genome assembly (GCA_963971225.1) was annotated by Ensembl at the European Bioinformatics Institute (EBI) using the BRAKER2 annotation system. This annotation includes 21 533 protein-coding genes. For further information about the annotation, please refer to the annotation page on Ensembl.

### Wellcome Sanger Institute – Legal and Governance

The materials that have contributed to this genome note have been supplied by a Darwin Tree of Life Partner. The submission of materials by a Darwin Tree of Life Partner is subject to the **‘Darwin Tree of Life Project Sampling Code of Practice’**, which can be found in full on the Darwin Tree of Life website. By agreeing with and signing up to the Sampling Code of Practice, the Darwin Tree of Life Partner agrees they will meet the legal and ethical requirements and standards set out within this document in respect of all samples acquired for, and supplied to, the Darwin Tree of Life Project. Further, the Wellcome Sanger Institute employs a process whereby due diligence is carried out proportionate to the nature of the materials themselves, and the circumstances under which they have been/are to be collected and provided for use. The purpose of this is to address and mitigate any potential legal and/or ethical implications of receipt and use of the materials as part of the research project, and to ensure that in doing so we align with best practice wherever possible. The overarching areas of consideration are:

- Ethical review of provenance and sourcing of the material
- Legality of collection, transfer and use (national and international)

Each transfer of samples is further undertaken according to a Research Collaboration Agreement or Material Transfer Agreement entered into by the Darwin Tree of Life Partner, Genome Research Limited (operating as the Wellcome Sanger Institute), and in some circumstances, other Darwin Tree of Life collaborators.

## Data Availability

European Nucleotide Archive: Melieria omissa. Accession number PRJEB67433. The genome sequence is released openly for reuse. The *Melieria omissa* genome sequencing initiative is part of the Darwin Tree of Life Project (PRJEB40665) and the Sanger Institute Tree of Life Programme (PRJEB43745). All raw sequence data and the assembly have been deposited in INSDC databases. Raw data and assembly accession identifiers are reported in Table 1 and Table 2. Production code used in genome assembly at the WSI Tree of Life is available at https://github.com/sanger-tol. Table 5 lists software versions used in this study.

## Author information

Contributors are listed at the following links:

- Members of the Natural History Museum Genome Acquisition Lab
- Members of the Darwin Tree of Life Barcoding collective
- Members of the Wellcome Sanger Institute Tree of Life Management, Samples and Laboratory team
- Members of Wellcome Sanger Institute Scientific Operations – Sequencing Operations
- Members of the Wellcome Sanger Institute Tree of Life Core Informatics team
- Members of the Tree of Life Core Informatics collective
- Members of the Darwin Tree of Life Consortium

## References

[ref-1] Allio R Schomaker-Bastos A Romiguier J : MitoFinder: efficient automated large-scale extraction of mitogenomic data in target enrichment phylogenomics. *Mol Ecol Resour.* 2020;20(4):892–905. 10.1111/1755-0998.13160 32243090 PMC7497042

[ref-2] Altschul SF Gish W Miller W : Basic Local Alignment Search Tool. *J Mol Biol.* 1990;215(3):403–410. 10.1016/S0022-2836(05)80360-2 2231712

[ref-3] Bateman A Martin MJ Orchard S : UniProt: the Universal Protein Knowledgebase in 2023. *Nucleic Acids Res.* 2023;51(D1):D523–D531. 10.1093/nar/gkac1052 36408920 PMC9825514

[ref-4] Buchfink B Reuter K Drost HG : Sensitive protein alignments at Tree-of-Life scale using DIAMOND. *Nat Methods.* 2021;18(4):366–368. 10.1038/s41592-021-01101-x 33828273 PMC8026399

[ref-5] Challis R Richards E Rajan J : BlobToolKit – interactive quality assessment of genome assemblies. *G3 (Bethesda).* 2020;10(4):1361–1374. 10.1534/g3.119.400908 32071071 PMC7144090

[ref-6] Chandler P : An update of the 1998 checklist of Diptera of the British Isles.[updated 23 may 2025].2025. Reference Source

[ref-7] Cheng H Concepcion GT Feng X : Haplotype-resolved *de novo* assembly using phased assembly graphs with hifiasm. *Nat Methods.* 2021;18(2):170–175. 10.1038/s41592-020-01056-5 33526886 PMC7961889

[ref-8] Clements DK : Provisional keys to the Otitidae and Platystomatidae of the British Isles. *Dipterists Digest.* 1990;6:32–41. Reference Source

[ref-9] Clements DK : Keys to British picture-wing flies (Diptera: Tephritidae, Ulidiidae, Platystomatidae, Pallopteridae and Opomyzidae).2020.

[ref-10] Colyer CN Hammond CO : Flies of the British Isles. London: Warne,1951. Reference Source

[ref-11] Crowley L Allen H Barnes I : A sampling strategy for genome sequencing the British terrestrial arthropod fauna [version 1; peer review: 2 approved]. *Wellcome Open Res.* 2023;8:123. 10.12688/wellcomeopenres.18925.1 37408610 PMC10318377

[ref-12] Danecek P Bonfield JK Liddle J : Twelve years of SAMtools and BCFtools. *GigaScience.* 2021;10(2): giab008. 10.1093/gigascience/giab008 33590861 PMC7931819

[ref-13] Ewels P Magnusson M Lundin S : MultiQC: summarize analysis results for multiple tools and samples in a single report. *Bioinformatics.* 2016;32(19):3047–3048. 10.1093/bioinformatics/btw354 27312411 PMC5039924

[ref-14] Ewels PA Peltzer A Fillinger S : The nf-core framework for community-curated bioinformatics pipelines. *Nat Biotechnol.* 2020;38(3):276–278. 10.1038/s41587-020-0439-x 32055031

[ref-15] Formenti G Abueg L Brajuka A : Gfastats: conversion, evaluation and manipulation of genome sequences using assembly graphs. *Bioinformatics.* 2022;38(17):4214–4216. 10.1093/bioinformatics/btac460 35799367 PMC9438950

[ref-16] GBIF Secretariat: *Melieria omissa* (Meigen, 1826) in GBIF Backbone Taxonomy. Checklist dataset.2023. Reference Source

[ref-17] Greve L : *Melieria omissa* (Meigen, 1826) and *Tetanops myopina*. *Fauna Norvegica SER B.* 1988;35(2):61. Reference Source

[ref-18] Howard C Denton A Jackson B : On the path to reference genomes for all biodiversity: lessons learned and laboratory protocols created in the Sanger Tree of Life core laboratory over the first 2000 species. *bioRxiv.* 2025. 10.1101/2025.04.11.648334 PMC12548527 41129326

[ref-19] Howe K Chow W Collins J : Significantly improving the quality of genome assemblies through curation. *GigaScience.* 2021;10(1): giaa153. 10.1093/gigascience/giaa153 33420778 PMC7794651

[ref-20] Kabos WJ van Aartsen B : De Nederlandse boorvliegen (Tephritidae) en prachtvliegen (Otitidae). *Wetenschappelijke Mededelingen van de Koninklijke Nederlandse Natuurhistorische Vereniging.* 1984;163:1–52. Reference Source

[ref-21] Kerpedjiev P Abdennur N Lekschas F : HiGlass: web-based visual exploration and analysis of genome interaction maps. *Genome Biol.* 2018;19(1): 125. 10.1186/s13059-018-1486-1 30143029 PMC6109259

[ref-22] Kurtzer GM Sochat V Bauer MW : Singularity: scientific containers for mobility of compute. *PLoS One.* 2017;12(5): e0177459. 10.1371/journal.pone.0177459 28494014 PMC5426675

[ref-23] Lawniczak MKN Davey RP Rajan J : Specimen and sample metadata standards for biodiversity genomics: a proposal from the Darwin Tree of Life project [version 1; peer review: 2 approved with reservations]. *Wellcome Open Res.* 2022;7:187. 10.12688/wellcomeopenres.17605.1

[ref-24] Li H : Minimap2: pairwise alignment for nucleotide sequences. *Bioinformatics.* 2018;34(18):3094–3100. 10.1093/bioinformatics/bty191 29750242 PMC6137996

[ref-25] Manni M Berkeley MR Seppey M : BUSCO update: novel and streamlined workflows along with broader and deeper phylogenetic coverage for scoring of eukaryotic, prokaryotic, and viral genomes. *Mol Biol Evol.* 2021;38(10):4647–4654. 10.1093/molbev/msab199 34320186 PMC8476166

[ref-26] Merkel D : Docker: lightweight Linux containers for consistent development and deployment. *Linux J.* 2014;2014(239): 2. Reference Source

[ref-27] Mitchell R Natural History Museum Genome Acquisition Lab, Darwin Tree of Life Barcoding collective : The genome sequence of a picture-winged fly, *Melieria crassipennis* (Fabricius, 1794) [version 1; peer review: 3 approved]. *Wellcome Open Res.* 2024;9:614. 10.12688/wellcomeopenres.23224.1 39554247 PMC11569385

[ref-28] NBN Atlas Partnership: *Melieria omissa* (Meigen, 1826).2025. Reference Source

[ref-29] O’Leary NA Cox E Holmes JB : Exploring and retrieving sequence and metadata for species across the Tree of Life with NCBI Datasets. *Sci Data.* 2024;11(1): 732. 10.1038/s41597-024-03571-y 38969627 PMC11226681

[ref-30] Ranallo-Benavidez TR Jaron KS Schatz MC : GenomeScope 2.0 and Smudgeplot for reference-free profiling of polyploid genomes. *Nat Commun.* 2020;11(1): 1432. 10.1038/s41467-020-14998-3 32188846 PMC7080791

[ref-31] Rao SSP Huntley MH Durand NC : A 3D map of the human genome at kilobase resolution reveals principles of chromatin looping. *Cell.* 2014;159(7):1665–1680. 10.1016/j.cell.2014.11.021 25497547 PMC5635824

[ref-32] Rhie A McCarthy SA Fedrigo O : Towards complete and error-free genome assemblies of all vertebrate species. *Nature.* 2021;592(7856):737–746. 10.1038/s41586-021-03451-0 33911273 PMC8081667

[ref-33] Rhie A Walenz BP Koren S : Merqury: reference-free quality, completeness, and phasing assessment for genome assemblies. *Genome Biol.* 2020;21(1): 245. 10.1186/s13059-020-02134-9 32928274 PMC7488777

[ref-34] Schoch CL Ciufo S Domrachev M : NCBI Taxonomy: a comprehensive update on curation, resources and tools. *Database (Oxford).* 2020;2020: baaa062. 10.1093/database/baaa062 32761142 PMC7408187

[ref-35] Smit JT : De prachtvlieg *Melieria picta* in grote aantallen op strandkweek *Elytrigia atherica* op Schiermonnikoog (Diptera: Ulidiidae). *Nederlandse Faunistische Mededelingen.* 2010;33:1–8. Reference Source

[ref-36] Smith KGV : An introduction to the immature stages of British flies.1989;10(14). Reference Source

[ref-37] Speight MCD Chandler PJ : Irish Otitidae & Platystomatidae (Diptera) including a key to the genera known in Ireland and/or Great Britain. *Ir Nat J.* 1983;21(3):130–36. Reference Source

[ref-38] Twyford AD Beasley J Barnes I : A DNA barcoding framework for taxonomic verification in the Darwin Tree of Life project [version 1; peer review: 2 approved]. *Wellcome Open Res.* 2024;9:339. 10.12688/wellcomeopenres.21143.1 39386966 PMC11462125

[ref-39] Uliano-Silva M Ferreira JGRN Krasheninnikova K : MitoHiFi: a python pipeline for mitochondrial genome assembly from PacBio high fidelity reads. *BMC Bioinformatics.* 2023;24(1): 288. 10.1186/s12859-023-05385-y 37464285 PMC10354987

[ref-40] Vasimuddin M Misra S Li H : Efficient architecture-aware acceleration of BWA-MEM for multicore systems.In: *2019 IEEE International Parallel and Distributed Processing Symposium (IPDPS).*IEEE,2019;314–324. 10.1109/IPDPS.2019.00041

[ref-41] Vicoso B Bachtrog D : Numerous transitions of sex chromosomes in Diptera. *PLoS Biol.* 2015;13(4): e1002078. 10.1371/journal.pbio.1002078 25879221 PMC4400102

[ref-42] Zhou C McCarthy SA Durbin R : YaHS: yet another Hi-C Scaffolding tool. *Bioinformatics.* 2023;39(1): btac808. 10.1093/bioinformatics/btac808 36525368 PMC9848053

