# Supplementary material for: The genome sequence of a picture-winged fly , Melieria omissa (Meigen, 1826) (Diptera: Ulidiidae)
Source: Wellcome Open Res. 2025 Oct 3;10:546. [Version 1] doi: 10.12688/wellcomeopenres.24956.1 (PMC12648027; doi:10.12688/wellcomeopenres.24956.1)
Supplement: Peer review history

# Reviewer report: Reviewer response for version 1

**DOI:** 10.21956/wellcomeopenres.27490.r136988

**Published:** 2025-11-25

**Authors:** Yu-Feng Huang

**Affiliations:** 1 National Chung Hsing University, Taichung City, Taichung City, Taiwan

This genome note reports a reference genome for the picture-winged fly *Melieria omissa* (Diptera: Ulidiidae), generated within the Darwin Tree of Life project, which aims to produce high-quality reference genomes for all eukaryotes in Britain and Ireland. An adult female collected from Berney Marshes, England, was taxonomically verified and used for whole-genome sequencing.

 High-molecular-weight DNA was extracted from whole-body tissue and sequenced with PacBio HiFi. A chromosome-scale assembly was produced using hifiasm and scaffolded with Hi-C data under the standardised DToL pipeline for assembly, curation, and quality control. The primary assembly is about 490 Mb, with most sequence assigned to four chromosomal pseudomolecules that match the haploid karyotype, and shows high continuity, completeness, and base-level accuracy by EBP metrics and BUSCO.

 The mitochondrial genome was assembled as a single circular molecule (~15.8 kb). Ensembl annotation identified over 21,000 protein-coding genes, providing a comprehensive gene catalogue for future analyses. All raw data and assemblies are openly available in INSDC repositories. Together, these resources offer a robust genomic foundation for studies of ulidiid biology, Tephritoidea phylogeny, and broader questions in insect genome evolution. However, several issues and limitations should be noted as follows:

1. This study forms part of the Darwin Tree of Life project, which follows a predefined, standardised workflow from specimen collection through sequencing, assembly, and analysis, leaving limited flexibility to deviate from the core protocol.
2. Because the Darwin Tree of Life pipeline is optimised for chromosome-scale nuclear genome assembly using PacBio HiFi and Hi-C data, the mitochondrial genome is handled as a small organellar assembly, with fewer opportunities for bespoke optimisation or curation.
3. The current genome-note format mainly reports assembly statistics and quality-control metrics, providing relatively little space for detailed biological interpretation of the mitochondrial genome and its functional features.

Are sufficient details of methods and materials provided to allow replication by others?

Yes

Is the rationale for creating the dataset(s) clearly described?

Yes

Are the datasets clearly presented in a useable and accessible format?

Yes

Are the protocols appropriate and is the work technically sound?

Yes

Reviewer Expertise:

Bioinformatics, Mitogenomics Analysis, Cancer Biology

I confirm that I have read this submission and believe that I have an appropriate level of expertise to confirm that it is of an acceptable scientific standard.

# Reviewer report: Reviewer response for version 1

**DOI:** 10.21956/wellcomeopenres.27490.r136987

**Published:** 2025-11-24

**Authors:** Stuart J.E. Baird

**Affiliations:** 1 Academy of Sciences of the Czech Republic, Národní, Staré Město, Czech Republic

The report adheres to the (excelent) format of a TreeOfLife genome sequence report.

 I have one suggestion:

 On page ten (top left) the number of protein-coding genes in the annotation is written with a space used for digit grouping. While a space is one generally accepted form of digit grouping, once the text is justified, the space becomes a very long space, which is not a generally recognised form of digit grouping.

 If a comma was used for digit grouping, the number of protein-coding genes would be written 21,533 (twenty one thousand five hundred and thirty three).

 The justified text presents this number as 

 "This annotation includes 21   533 protein-coding genes"

 ...potentially leaving the reader asking what are 533 coding genes, and why are there only 21 of them?

Are sufficient details of methods and materials provided to allow replication by others?

Yes

Is the rationale for creating the dataset(s) clearly described?

Yes

Are the datasets clearly presented in a useable and accessible format?

Yes

Are the protocols appropriate and is the work technically sound?

Yes

Reviewer Expertise:

Admixture genomics, spatial genetics

I confirm that I have read this submission and believe that I have an appropriate level of expertise to confirm that it is of an acceptable scientific standard.
